# EB病毒阳性血管内大B细胞淋巴瘤1例

**DOI:** 10.3760/cma.j.issn.0253-2727.2023.10.016

**Published:** 2023-10

**Authors:** 晓娜 左, 柳倚 杨, 璐 孙, 丽改 郭, 子芬 高

**Affiliations:** 1 北京高博博仁医院病理科，北京 100071 Department of Pathology, Beijing GoBroad Boren Hospital, Beijing 100071, China; 2 解放军总医院第一医学中心病理科，北京 100853 Department of Pathology, the First Medical Center of Chinese PLA General Hospital, Beijing 100853, China; 3 北京大学第三医院病理科，北京 100191 Department of Pathology, Peking University Third Hospital, Beijing 100191, China

患者，女，25岁。患者于2022年9月发现颈部左侧有一约1.3 cm×0.8 cm大小肿块，质硬，无触痛，活动性差，就诊于当地医院，行颈部超声示：左颈部Ⅱ区可见3.8 cm×2.8 cm×1.6 cm实性肿物，予口服及静脉应用抗生素后未见好转。患者10月12日行超声引导下肿物穿刺，术后病理示：（颈部左侧肿物）淋巴组织增生。患者2022年10月就诊于解放军总医院第一医学中心，行PET/CT检查，结果示：颈部左侧血管旁多发增大淋巴结，FDG摄取异常增高，最大标准摄取值（SUVmax）＝23.1，病变边界不清，与相邻左侧颌下腺、左侧胸锁乳突肌分界不清，PET/CT融合图显示大者约3.4 cm×2.7 cm×3.6 cm，病变密度尚均匀，CT值约60 Hu。双侧锁骨区、双侧腋窝、双侧腹股沟区未见明显肿大淋巴结。患者2022年11月行颈部左侧肿物切除术，术中送检（左颈Ⅱ区）淋巴结5枚，大者1.0 cm×0.7 cm×0.5 cm，小者0.2 cm×0.2 cm×0.2 cm。术后送检（左颈Ⅱ区）淋巴结3枚，大者4.5 cm×4.0 cm×1.5 cm，部分被膜破损，切面灰红色及灰白色，质地中等；小者0.3 cm×0.2 cm×0.2 cm，质地中等。病理示：（左颈Ⅱ区）淋巴结B细胞源性淋巴瘤，考虑血管内大B细胞淋巴瘤可能性大。

2022年11月患者于北京高博博仁医院行病理会诊，显微镜下可见大小不等淋巴结多枚，小淋巴结被膜薄，边缘窦开放，淋巴结结构大致正常，T区细胞混合性增生，淋巴窦可见。大淋巴结被膜增厚、纤维化，边缘见残留的淋巴滤泡，T区明显增宽，见灶状上皮样细胞增生，似肉芽肿样结构，伴小淋巴细胞及浆细胞浸润。近边缘区域见数量不等的脉管，脉管内可见明显异常细胞聚集，异常细胞体积中等或略偏大，胞质少，核类圆，染色质细颗粒状，少量见核仁（图1），肿瘤细胞面积约占淋巴结总面积的5％。免疫组化：B细胞标志CD20、PAX5、BOB1、OCT2阳性，证明为B细胞；细胞起源标志BCL6、CD10阳性，提示为生发中心B细胞起源；增殖活性Ki-67约90％；T细胞标志物CD3、CD4、CD5、CD8均阴性，除外了T细胞分化；树突状细胞网CD21、CD23阴性；其他标志：C-myc约40％阳性，CD34、成红细胞转化特异性相关基因（ERG）血管内皮细胞阳性，进一步证明肿瘤细胞全部位于小血管内；CD163、CyclinD1、TDT肿瘤细胞均阴性。EB病毒编码的小mRNA（EBER）肿瘤细胞70％阳性（图1）。

**图1:**
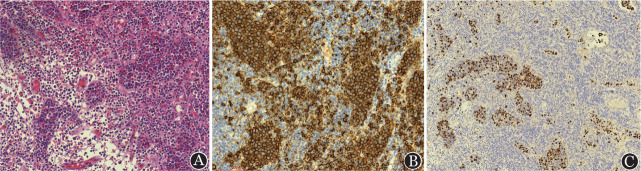
患者颈部左侧Ⅱ区淋巴结HE染色及免疫组织化学染色 A HE染色（×200）； B CD20血管内肿瘤细胞阳性，周围小淋巴细胞阳性（×200）； C EB病毒编码的小mRNA，血管内肿瘤细胞大部分阳性（×100）

石蜡组织切片行PCR-IG克隆性分析检测，IGH-A、IGH-B、IGH-C、IGH-E阳性，提示增生细胞为单克隆性，支持为B细胞肿瘤。应用MYC、BCL2、BCL6分离探针对石蜡组织切片进行FISH检测，检测到MYC基因分离阳性信号（信号模式：1红1绿1融合，图2），BCL2、BCL6未见典型基因分离阳性信号（正常信号模式均为2融合）。B系淋巴瘤相关基因突变筛查（二代测序法，共检测163个基因，覆盖度>99％，平均测序深度1500×以上）检测到ID3 p.Q81X、FAT1 p.K1259Sfs*17及MYC p.T73I变异。

**图2:**
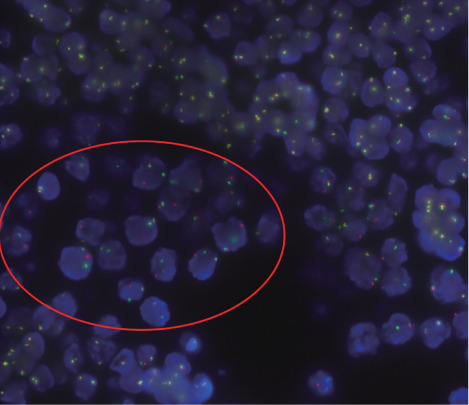
FISH显示患者肿瘤细胞MYC基因分离阳性 注 应用MYC断裂探针进行检测（正常信号为2个红色2个绿色融合性信号，颜色略显黄色），图中椭圆形红圈内区域为肿瘤细胞所在区域，可见典型阳性信号（1红1绿1融合信号），其余区域细胞未见阳性分离信号

2022年12月患者为进一步治疗转入北京高博博仁医院。血常规：WBC 5.80×10^9^/L，HGB 137 g/L，PLT 230×10^9^/L。全身浅表淋巴结彩超示：颈部双侧可探及多发淋巴结，形态规则，髓质结构尚可分辨，较大者位于Ⅱ区，右侧大小约1.3 cm×1.0 cm×0.5 cm，左侧大小约1.5 cm×0.8 cm×0.6 cm。双侧锁骨区、双侧腋窝、双侧腹股沟未见明显肿大淋巴结。行骨髓穿刺及腰椎穿刺，骨髓液细胞形态学及流式细胞术、脑脊液流式细胞术检查均未见异常B细胞。予R-CODOX-M（利妥昔单抗+环磷酰胺+长春新碱+阿霉素+地塞米松+甲氨蝶呤）/IVAC方案（异环磷酰胺+依托泊苷+阿糖胞苷）规律化疗4个周期，2023年3月患者行PET-CT检查未见明确肿瘤活性征象，评估为完全缓解。患者于2023年7月结束全部8个周期治疗，目前定期随访观察中。

讨论：血管内大B细胞淋巴瘤是结外大B细胞淋巴瘤的罕见类型，但WHO分类中并未提及肿瘤细胞是否与EB病毒感染相关，仅有少数病例报道显示肿瘤细胞EBER原位杂交阳性。本例肿瘤细胞体积中等或略偏大，为生发中心起源，BCL2蛋白水平阴性，细胞增殖活性高，与伯基特淋巴瘤形态学及免疫表型类似，且EB病毒阳性，FISH检测到MYC分离阳性、分子二代测序检测到较常出现于伯基特淋巴瘤中的ID3、MYC等基因突变。有文献报道ID3基因突变在34％～68％的伯基特淋巴瘤中出现，也曾在双重打击淋巴瘤等中检出，在弥漫大B细胞淋巴瘤中少见。且本例患者MYC p.T73I变异为非同义突变，不是伯基特淋巴瘤中常见的重排、错义变异。在这种情况下，不应仅凭遗传及分子生物学结果给出伯基特淋巴瘤的诊断，应该回归到WHO分类中淋巴瘤类型的形态学特点，本例患者显微镜下观察到瘤细胞全部位于血管内，是血管内大B细胞淋巴瘤非常重要的特点和鉴别点。

